# Bonding Pretreatment of Aesthetic Dental CAD-CAM Materials through Surface Etching with a Mixed Aqueous Solution of Ammonium Fluoride and Ammonium Hydrogen Sulfate

**DOI:** 10.3390/jfb15030071

**Published:** 2024-03-14

**Authors:** Yusaku Nishizawa, Tatsuo Kawamoto, Hiroshi Ikeda

**Affiliations:** 1 Division of Biomaterials, Department of Oral Functions, Kyushu Dental University, Kitakyushu 803-8580, Japan; 2 Division of Orofacial Functions and Orthodontics, Department of Health Improvement, Kyushu Dental University, Kitakyushu 803-8580, Japan; r15kawamoto@fa.kyu-dent.ac.jp

**Keywords:** fluoride, bond strength, dental material, luting agent, surface treatment, chemical etching, adhesion

## Abstract

Hydrofluoric acid (HF) is commonly used as an etchant for the pretreatment of dental computer-aided design/computer-aided manufacturing (CAD-CAM) materials, such as glass-ceramics and resin composites. Despite its effectiveness, the harmful and hazardous nature of HF has raised significant safety concerns. In contrast, ammonium fluoride (AF) is known for its relatively low toxicity but has limited etching capability. This study explored the potential of ammonium hydrogen sulfate (AHS), a low-toxicity and weak acid, to enhance the etching ability of aqueous AF solutions for the bonding pretreatment of CAD-CAM materials. This study investigated five types of aesthetic CAD-CAM materials: lithium disilicate glass, feldspathic porcelain, polymer-infiltrated ceramic networks, resin composites, and zirconia. Seven experimental etchants were prepared by varying the amount of AHS added to aqueous AF solutions, with each etchant used to etch the surfaces of the respective CAD-CAM materials. The treated surfaces were analyzed using scanning electron microscopy and confocal laser scanning microscopy. Additionally, the shear bond strength (SBS) of the CAD-CAM materials treated with a luting agent (resin cement) was evaluated. The results indicated that the AF1/AHS3 (weight ratio AF:AHS = 1:3) etchant had the most substantial etching effect on the surfaces of silica-containing materials (lithium disilicate glass, feldspathic porcelain, polymer-infiltrated ceramic networks, and resin composites) but not on zirconia. The SBS of the materials treated with the AF1/AHS3 etchant was comparable to that of the commercial HF etchant. Hence, an AF/AHS mixed solution could effectively etch silica-containing CAD-CAM materials, thereby enhancing their bonding capabilities.

## 1. Introduction

Computer-aided design computer-aided manufacturing (CAD-CAM) technology has significantly advanced dental restorations by enabling a more precise and rapid production of restorations [1,2]. This process involves scanning the prepared tooth, designing the restoration using CAD software, and fabricating it using CAM milling machines. Various types of tooth-colored aesthetic materials have been developed for this purpose, including ceramic-based and resin–ceramic hybrid (composite) materials [3,4]. In ceramics, subcategories such as polycrystalline ceramics and glass ceramics exist. Zirconia, a polycrystalline ceramic known for its strength and fracture toughness, is ideal for high-stress areas such as molar crowns and bridges [5,6]. Representative glass ceramics, such as lithium disilicate glass and feldspathic porcelain, are valued for their aesthetic and mechanical properties [5,7]. The resin–ceramic hybrid category encompasses materials such as resin composites and polymer-infiltrated ceramic networks. Resin composites consist of ceramic fillers embedded within a resin matrix, and recent advancements have made them less abrasive and more tooth-friendly, offering a balance between durability and gentleness during occlusion [8,9]. In contrast, polymer-infiltrated ceramic networks feature a unique dual-network structure that combines the ceramic and polymer phases. This innovative composition allows them to closely mimic the mechanical properties of human teeth, making them suitable for dental restorative applications [10,11]. These aesthetic CAD-CAM materials are known for their advantageous properties. However, they exhibit brittleness, potentially leading to fractures in clinical settings [12,13]. Thus, ensuring a strong bond between the material and the abutment tooth is essential to reduce this risk, as effective bonding reduces the likelihood of fractures and helps prevent debonding of materials [14,15,16]. Therefore, achieving durable and reliable bonding is crucial for the long-term success of tooth restorations using the aesthetic CAD-CAM materials.

For dependable bonding, surface pretreatment of CAD-CAM materials before applying a luting agent is vital for enhancing bond strength [17]. Extensive research has been conducted to improve the surface treatment methods for these materials. Techniques such as plasma irradiation [18,19], sandblasting [20,21], tribochemical coating [22,23], laser abrasion [24,25], and chemical etching [26,27] have been examined for their ability to modify material surfaces for better bonding with luting agents. Among these methods, chemical etching with hydrofluoric acid (HF) is notable for its efficiency in etching silica-containing materials [17,28] such as lithium disilicate glass, feldspathic porcelain, polymer-infiltrated ceramic networks, and resin composites. HF reacts with silica in these materials, roughening their surface, which increases the surface area and enhances mechanical interlocking at the material–luting agent interface, leading to improved bonding properties. Consequently, HF etching is recognized as the gold standard for the surface pretreatment of silica-containing materials, given its proven effectiveness in preparing these materials for strong and reliable bonding in dental restorations [29].

While HF is recognized for its effective etching of silica-containing materials, its substantial toxicity and related health hazards pose significant concerns, consequently resulting in an ongoing search for safer and less toxic alternatives that can potentially replace HF. Various candidates, including substances like phosphoric acid [30,31,32] and fluoride-based products such as Monobond Etch & Prime^®^ [33,34,35], have been explored for their suitability as surface conditioners. However, despite their reduced hazard profiles, these alternatives fall short of HF etching performance and display notably weaker etching capabilities [30,31,32,33,34,35]. Currently, there is no alternative etchant that can match the effectiveness of HF for chemically etching CAD-CAM surfaces, thus presenting a significant challenge in dentistry and highlighting the need to balance efficient surface conditioning with the use of safer and less harmful substances.

Hence, we focused on aqueous fluoride solutions to develop an alternative etchant to HF etchants. Previous studies have explored the etching effects of various aqueous fluoride solutions on glass ceramics and their impact on bonding with luting agents [36]. These findings indicate that aqueous ammonium hydrogen fluoride (ammonium bifluoride) solutions etched the surfaces of the materials (lithium disilicate glass, porcelain, and polymer-infiltrated ceramic networks) and improved their bond strength with the luting agent. Furthermore, other studies have validated the etching capabilities of ammonium bifluoride on glass ceramics [37] and zirconia [38]. Thus, ammonium bifluoride has emerged as a promising substitute for HF. However, despite its lower toxicity compared to HF, concerns regarding the toxicity of ammonium bifluoride in humans persist. Thus, the focus of this study shifted to ammonium fluoride (AF), known to exhibit an even lower toxicity. Although AF is less toxic and corrosive than ammonium bifluoride, AF cannot naturally etch CAD-CAM materials [36]. The solubility of silica in aqueous fluoride solutions is known to vary with pH, increasing as the pH decreases [39], suggesting that the etching capacity of AF solutions can be enhanced by adjusting the pH. Therefore, this study aimed to investigate whether the addition of an acid to AF solutions can enhance their etching effect on CAD-CAM materials and subsequently improve the bond strength between the material and the luting agent. Ammonium hydrogen sulfate (AHS), chosen for its low toxicity, was used to prepare various aqueous solutions combining AF and AHS. These solutions were tested for their etching abilities using different CAD-CAM materials. This study tested two null hypotheses: (1) the etching abilities of AF solutions are not influenced by the addition of AHS, and (2) the mixed solution of AF and AHS does not enhance the bond strength of CAD-CAM materials to a luting agent.

## 2. Materials and Methods

### 2.1. Experimental Etchants

The experimental etchants were prepared using AF, AHS, and distilled water, as detailed in Table 1. The composition of each experimental etchant is listed in Table 2. A specific amount of AF was added to distilled water and stirred continuously using a magnetic stirrer at a constant temperature of 25 °C. Subsequently, AHS was gradually added to the solution, with the stirring process maintained to ensure the complete dissolution of the substances, ultimately resulting in a homogenous solution. The prepared solutions served as experimental etchants for subsequent experiments. For comparison, a commercially available HF etchant was also used (Table 3). The pH values of the experimental etchants were determined using a pH meter (LAQUA F-72, Horiba Ltd., Kyoto, Japan) at 25 °C.

### 2.2. CAD-CAM Materials

Table 4 presents commercially available dental restorative materials, including lithium disilicate glass, feldspathic porcelain, polymer-infiltrated ceramic network, resin composite, and zirconia. These materials are typically used in CAD-CAM applications. Each material was sectioned into plates with a thickness of 2 mm using a diamond wheel saw (MINICUT 40, SCAN-DIA GmbH & CO. KG, Hagen, Germany), with water cooling employed during the cutting process. The surfaces of these plates were sequentially polished using emery papers of increasing fineness: #400, #1000, and #2000 grits. Following the polishing process, the plates underwent ultrasonic cleaning in distilled water for 5 min and were dried using an air blower. These cleaned plates were then utilized in the experimental procedures.

### 2.3. Etching Procedure

The chemical etching treatment using the experimental etchants was performed as follows: A total of 20 μL of each etchant was applied to the surface of the CAD-CAM material using a micropipette. The samples were then left to sit undisturbed for 60 s at a steady temperature of 25 °C in ambient conditions. Following this incubation period, each sample was thoroughly rinsed under running water for 15 s to eliminate any remaining etchants from the surface. After rinsing, the samples were cleaned ultrasonically in water for 5 min. After the cleaning, the samples were dried using an air blower. The prepared samples were subsequently utilized for further characterization in subsequent experiments.

### 2.4. Scanning Electron Microscopy (SEM) Observation

The etchant-treated samples were analyzed using scanning electron microscopy (SEM; model JCM-7000, JEOL, Tokyo, Japan) at an acceleration voltage of 15 kV. Before the observations, the surfaces of the samples were sputter-coated with platinum to enhance the image quality.

### 2.5. Confocal Laser Scanning Microscopy Analysis

The surfaces of the samples were examined by confocal laser scanning microscopy (CLSM; model VKX-100, Keyence, Osaka, Japan) at ×50. The surface roughness parameter, Ra, was determined from the CLSM images using analytical software. Five separate measurements were taken for each sample (*n* = 5) to calculate the mean Ra value.

### 2.6. Shear Bond Strength (SBS) Test

**Figure 1:**
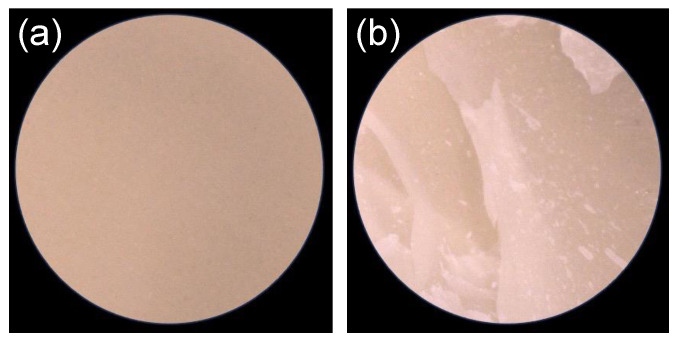
Typical failure mode of the cement-debonded sample surfaces after the shear bond strength test; (**a**) adhesive failure and (**b**) cohesive failure. The surfaces were observed using the optical microscope with 30× magnification.

The experimental etchant with the highest etching capacity was selected to investigate its effect on the bond strength of the luting agent. Commercial HF was used as a reference standard. The methodology for the SBS test was based on a protocol detailed in a previous study [40]. Each etchant-treated sample was secured in an acrylic tube using an autocured resin. A Teflon tube with an inner diameter of 5 mm was affixed to the etched surface of each sample using double-sided tape to ensure a consistent bonding area of 19.6 mm². Subsequently, a commercial adhesive primer (Table 3) was applied to the sample surfaces according to the manufacturer’s instructions. Subsequently, a layer of commercial resin cement (listed in Table 3) was applied over the primer-treated surface at a height of 2 mm. This layer was cured using a light irradiator (α Light II N, J. Morita, Osaka, Japan) for 5 min, followed by a stabilization period of 1 h at 25 °C. After removing the Teflon tube and tape, the cement-bonded samples were immersed in distilled water at 37 °C for 24 h. Using a thermocycling machine (K178, Tokyo Giken, Tokyo, Japan), the samples were subjected to accelerated aging, which included 20,000 thermocycles alternating between water baths at 5 °C and 55 °C, with each cycle lasting 60 s. The SBSs of the samples with and without accelerated aging were determined using a mechanical testing machine (AGS-H, Shimadzu, Kyoto, Japan) at a crosshead speed of 1.0 mm/min (*n* = 7). Post-SBS test, the cement-debonded surfaces were analyzed using optical microscopy. The observed failure modes were classified into two categories: adhesive failures at the cement–sample interface and cohesive failures within the sample (Figure 1).

### 2.7. Statistical Analysis

The data collected, specifically Ra and SBS, were analyzed using EZR statistical software (EZR version 1.62, Saitama Medical Center, Jichi Medical University, Saitama, Japan). For multiple comparisons, a one-way analysis of variance (ANOVA) was performed, followed by Tukey’s post hoc test for pairwise comparisons. Statistical significance was set at a significance level of 0.05, which served as the standard threshold.

## 3. Results

**Figure 2:**
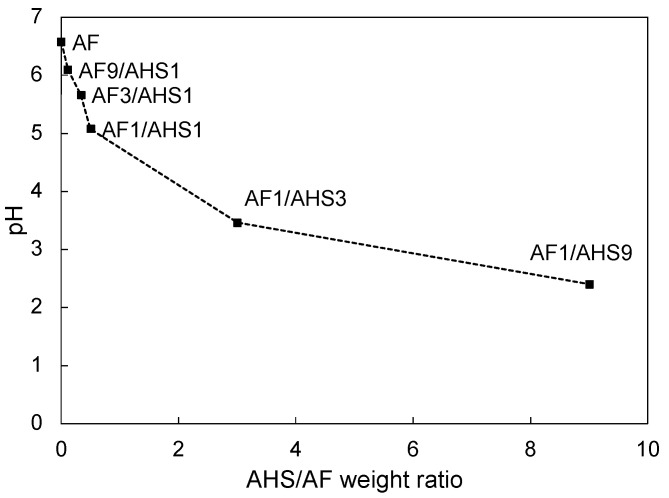
Relationship between pH values and weight ratio of ammonium fluoride (AF) and ammonium hydrogen sulfate (AHS) in experimental etchant.

Figure 2 shows the pH values of the various experimental etchants. As the concentration of AHS in the etchant increases, there is a corresponding decrease in pH. Specifically, the AF1/AHS3 and AF1/AHS9 etchants exhibit pH levels of 3.5 and 2.4, respectively. The AHS etchant, which does not contain AF, had a pH of 0.7. These results indicate a clear correlation between the proportion of AHS in the mixture and the pH of the etchant.

**Figure 3:**
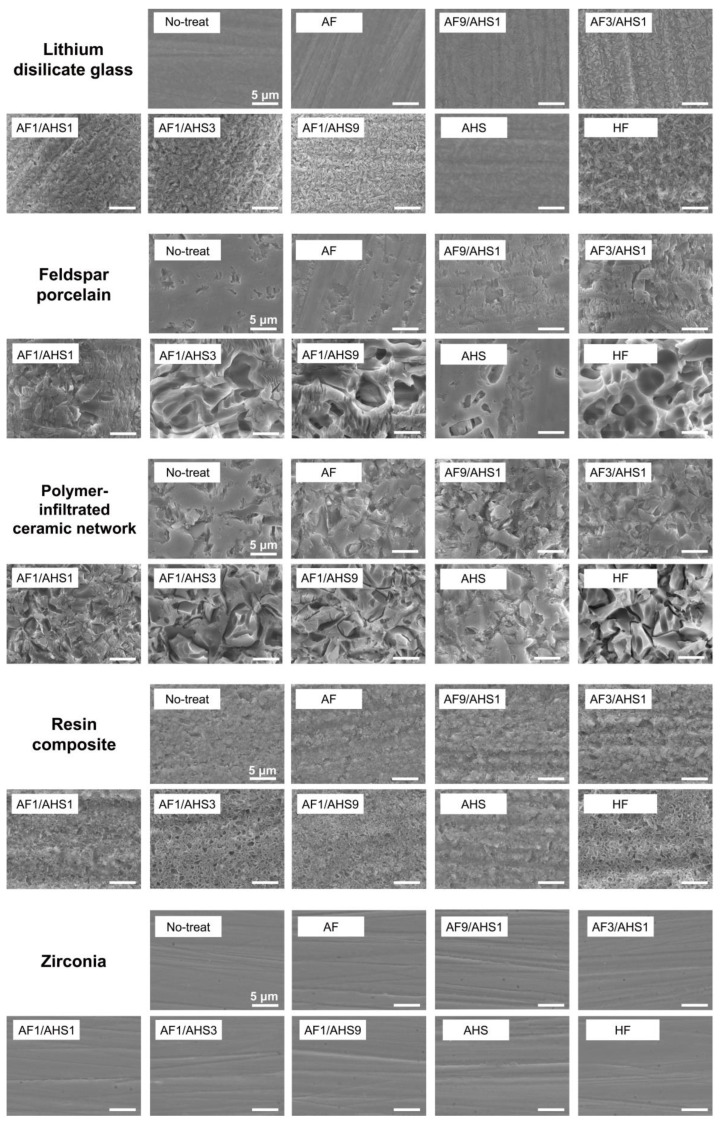
SEM images for CAD-CAM material surfaces after treatment with experimental etchants and commercial HF etchant.

Figure 3 displays SEM images of various material surfaces treated with the etchants, showing that the treatments increased the surface roughness of materials such as lithium disilicate glass, feldspathic porcelain, polymer-infiltrated ceramic networks, and resin composites, with a more pronounced etching effect observed at higher AHS concentrations. In particular, the AF1/AHS3 and AF1/AHS9 etchants exposed needle-like crystals on the lithium disilicate glass and significantly roughened the surfaces of the feldspathic porcelain and polymer-infiltrated ceramic network by removing the glass components; the resin composite surfaces were roughened because of the removal of the filler components. However, no etching effect was noted on any material surface treated solely with AF and AHS etchants. For zirconia, none of the etchants, including the AF1/AHS3 and AF1/AHS9 etchants, or the commercial HF etchant were able to roughen the surface, indicating a distinct resistance of this material to the etching process used.

**Figure 4:**
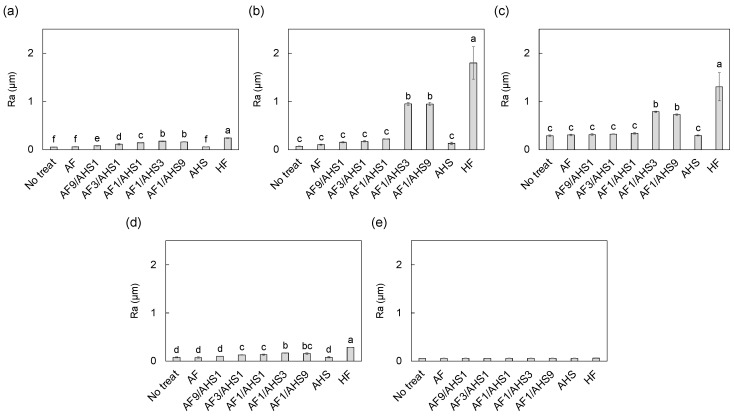
Ra values for CAD-CAM material surfaces after treatment with experimental etchants or commercial HF etchant: (**a**) lithium disilicate glass, (**b**) feldspathic porcelain, (**c**) polymer-infiltrated ceramic network, (**d**) resin composite, and (**e**) zirconia. The different alphabetic letters in the figure indicate significant differences between the groups (*p* < 0.05, Tukey’s test).

The quantitative etching effects of the experimental etchants on the CAD-CAM materials were evaluated using the Ra values obtained from CLSM, as shown in Figure 4. These results were consistent with the SEM observations. A clear trend was observed, where the Ra values of each CAD-CAM material, including lithium disilicate glass, feldspathic porcelain, the polymer-infiltrated ceramic network, and the resin composite, increased as the concentration of AHS in the etchant increased. Notably, the highest Ra values for each material were recorded when treated with AF1/AHS3 or AF1/AHS9 etchants, indicating the most significant surface roughening by these formulations. In contrast, AF and AHS etchants did not effectively etch any of the materials. Remarkably, none of the etchants, including those with the highest AHS concentrations, increased the Ra value of zirconia, underscoring its resistance to the etching process.

**Figure 5:**
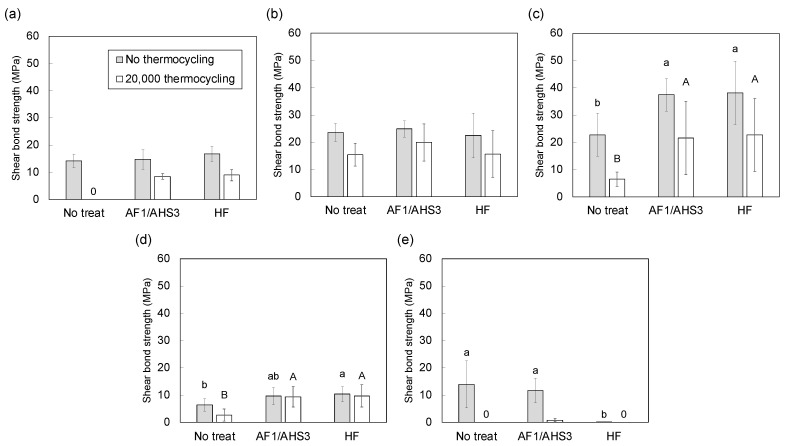
SBS values for CAD-CAM material surfaces treated with the experimental AF1/AHS3 etchant or commercial HF etchant: (**a**) lithium disilicate glass, (**b**) feldspathic porcelain, (**c**) polymer-infiltrated ceramic network, (**d**) resin composite, and (**e**) zirconia. The different alphabetic letters in the figure indicate significant differences between the groups (*p* < 0.05, Tukey’s test).

The AF1/AHS3 etchant, which demonstrated the best etching effect among the experimental etchants, was further investigated for its effect on the bonding of various CAD-CAM materials. Figure 5 shows the SBS between the luting agent and treated CAD-CAM materials. For context, comparisons were made with the SBSs for non-treated and HF-treated materials. The results revealed that the AF1/AHS3 etchant significantly enhanced the SBS of all the materials tested, except for zirconia. Specifically, the SBS values for the AF1/AHS3-treated samples were substantially higher than those of their non-treated counterparts and comparable to those of the HF-treated sample. Meanwhile, the SBS values of the AF1/AHS3-treated samples decreased following thermocycling. This decline in SBS post-thermocycling was noted for all treated materials but remained higher than that of the non-treated samples and equivalent to the HF-treated ones. These findings suggest that the AF1/AHS3 etchant effectively improves the bond strengths of lithium disilicate glass, feldspathic porcelain, polymer-infiltrated ceramic networks, and resin composites.

The failure modes of the SBS-tested samples are listed in Table 5. No differences were noted in the failure modes among the non-treated, AF1/AHS3, and HF-treated samples in the lithium disilicate glass, feldspathic porcelain, resin composite, and zirconia groups. In the polymer-infiltrated ceramic network groups, the number of cohesive failure samples was greater in the samples treated with the AF1/AHS3 etchant than in the untreated samples, implying that the AF1/AHS3-treated material was well bonded with the luting agent.

## 4. Discussion

This study investigated the surface etching effects of a mixed solution of AF and AHS (an experimental etchant) on various aesthetic CAD-CAM materials for bonding pretreatment. The findings revealed that the AF1/AHS3 etchant effectively etched several CAD-CAM materials, such as lithium disilicate glass, feldspathic porcelain, polymer-infiltrated ceramic networks, and resin composites, but did not affect zirconia. The CAD-CAM materials treated with the AF1/AHS3 etchant exhibited higher SBS than their non-treated counterparts. Consequently, we partially reject the first null hypothesis that the etching abilities of the AF solutions are not affected by the addition of AHS. Furthermore, the second null hypothesis, i.e., the mixed solution of AF and AHS (experimental etchant) does not improve the bond strength of CAD-CAM materials to a luting agent, was partially rejected.

The CAD-CAM materials used in this study were categorized into silica- and non-silica-containing materials. Silica-containing materials include lithium disilicate glass, feldspathic porcelain, polymer-infiltrated ceramic networks, and resin composites. Lithium disilicate glass is a high-strength glass ceramic composed of lithium disilicate crystals within a silicate glass matrix [5]. Feldspathic porcelain is a traditional glass ceramic comprising silicate glass and mineral crystals [5]. Polymer-infiltrated ceramic networks are hybrid materials that combine porous silicate glass with infiltrated acrylic resin [5]. Resin composites comprise silica and silicate glass fillers within the resin matrix. Conversely, the zirconia used in this experiment primarily consisted of 3 mol% Y_2_O_3_ stabilized tetragonal zirconia polycrystalline (3Y-TZP) and was classified as a non-silica-containing material [5]. The present results showed that the experimental etchants could etch silica-containing materials without affecting the non-silica-containing material (zirconia). The etching ability of the experimental etchant was similar to that of a commercial HF etchant. For HF, the etching mechanism for silica involves HF attacking the siloxane networks, with the overall reaction described as SiO_2_ + 6HF → H_2_SiF_6_ + 2H_2_O [41]. In aqueous HF solutions, fluoride species such as F-, HF, (HF)_2_, and HF_2_^−^ are present, with HF_2_^−^ being particularly effective at breaking Si–O–Si bonds in vitreous SiO_2_ [41,42,43,44]. The reactivity of these species varies with the fluoride type, concentration, and solution pH. Notably, HF_2_^−^ increases as pH decreases, particularly between pH 3 and 4, enhancing etching capacity in acidic environments [44]. Applying these principles in the present results, the experimental etchant containing AF forms F^−^ in a neutral pH environment. Adding AHS lowered the pH, thus increasing the concentration of reactive HF_2_^−^ species, particularly at a pH of 3.5 (as shown in the AF1/AHS3 etchant). Consequently, the AF1/AHS3 etchant effectively roughened the surface of silica-containing materials, as demonstrated in this study. On the other hand, neither the AF1/AHS3 etchant nor the HF etchant were able to etch the zirconia surface. This can be attributed to the high chemical resistance of zirconia. Consistent with these findings, it has been reported that zirconia exhibits a high level of chemical resistance to hydrofluoric acid [45].

The present study employed Ceramic Primer Plus^®^ (Table 3) as an adhesive primer for bonding resin cement (luting agent) to various CAD-CAM materials. This adhesive primer contains two key functional monomers, γ-MPTS and MDP, both of which play critical roles in enhancing bond strength and contributing to the longevity and durability of the bonding. γ-MPTS, a well-known silane coupling agent used in several commercial adhesive primers [46], initiates the bonding process by undergoing hydrolysis, reacting with water to form silanol (Si-OH) groups. These groups subsequently condensed with the silanol groups on the material surface, establishing a chemical bond. The other end of the silane molecule, which typically incorporates a methacrylate group, was designed to react with resin-based materials. The methacrylate group copolymerizes with the acrylic resin in the cement, forming a covalent bond between the resin and silane. Consequently, silica-containing materials, such as glass-ceramics and resin composites, effectively bond with resin cement through γ-MPTS. This silane coupling agent mechanism can be applied to lithium disilicate glass, feldspathic porcelain, polymer-infiltrated ceramic networks, and resin composites. Additionally, Ceramic Primer Plus includes an MDP monomer that specifically targets the bonding of polycrystalline ceramics. MDP, a phosphate ester monomer, comprises methacrylate and phosphate groups. The methacrylate group enables copolymerization with other methacrylate resins in the resin cement, whereas the phosphate group exhibits a high affinity for polycrystalline ceramics such as alumina and zirconia [47]. MDP monomers chemically bonded to zirconia surfaces and reacted with the oxide layer. Zirconia surfaces contain Zr-OH groups that react with the P-OH groups in the MDP molecules, forming hydrogen bonds. Additionally, ionic bonds were formed between the P-O^−^ groups in MDP and Zr^+^ on the zirconia surface, highlighting the role of surface OH groups in zirconia as active sites for bonding with resin cement via MDP. However, the SBS values of the samples significantly decreased after thermocycling, possibly attributed to the inability of HF or the experimental etchants to adequately roughen the surface of the zirconia, thereby preventing effective mechanical interlocking at the interface between the resin cement and zirconia.

Among the various experimental etchants tested, the AF1/AHS3 etchant demonstrated the most significant etching effect on the CAD-CAM materials, except for zirconia. The effect of the AF1/AHS3 etchant on the SBS between the etched CAD-CAM materials and the luting agent was comparable to that achieved with the commercial HF etchant, indicating that the AF1/AHS3 etchant effectively increases the surface roughness and area of the CAD-CAM materials, enhancing their potential for mechanical interlocking at the interface between the luting agent and CAD-CAM materials. Such increases in the surface roughness and area of the material are crucial for creating a strong bond between these two components. These findings suggest that the experimental AF1/AHS3 etchant is a viable option for the pre-bonding treatment of CAD-CAM materials, excluding zirconia.

The AF1/AHS3 etchant appears to offer a lower-risk profile than the HF etchant, especially when considering its classification under the Globally Harmonized System of Classification and Labelling of Chemicals (GHS). HF is categorized as either Category 1 or Category 2 in terms of oral toxicity, highlighting its extreme toxicity and corrosive nature. In contrast, AF and AHS, the constituents of the AF1/AHS3 etchant, were deemed to have relatively low toxicity levels and were classified as less than Category 3. Additionally, the total fluorine concentration in the AF1/AHS3 etchant used in this study was formulated as a dilute aqueous solution with a concentration of approximately 2.5%. This total fluorine concentration is notably lower than that found in commercial HF etchants (approximately 9%). However, comprehensive biosafety assessments for AF1/AHS3 etchants have not been conducted to date. The potential toxicity of the combined effects of AF and AHS remains a concern and should not be overlooked. Given these considerations, future research including animal studies is crucial for a thorough evaluation of the biosafety of the AF1/AHS3 etchant. Such investigations will be vital in determining the practicality of these etchants as safer alternatives to HF without compromising their effectiveness in dental applications.

In the field of dental materials, the formulation of commercial etchants often involves adding thickening agents such as polyvinyl alcohol and polyethylene glycol [48]. These additives are commonly used to modify etchant consistency to enhance their ease of use and applicability in clinical settings. However, these thickening agents, while improving user-friendliness, can sometimes adversely affect the etching process of glass ceramics as well as the subsequent bond formation between the glass ceramics and the luting agent [32]. This interaction poses a potential concern regarding the effectiveness and reliability of the etching and bonding processes. In contrast to commercially available etchants, the experimental etchants employed in this study did not contain thickening agents. This absence raises an intriguing point of consideration regarding the potential effect of combining thickening agents with experimental etchants, specifically disputing how the inclusion of such agents influences the etching efficiency and bonding strength when using experimental etchants. In the present experiment, the untreated zirconia demonstrates stronger adhesion to the luting agent compared to the HF-treated one. This may be due to the adverse effect of the thickening agent in the etchant. The potential influence of thickening agents on etching and bonding efficiency warrants further investigation.

## 5. Conclusions

This study explored the efficacy of an experimental etchant, a mixture of AF and AHS, for the surface etching of various CAD-CAM dental materials. The findings revealed that the acidic AF1/AHS3 etchant effectively increased the surface roughness of silica-containing CAD-CAM materials, such as lithium disilicate glass, feldspathic porcelain, polymer-infiltrated ceramic networks, and resin composites, thereby enhancing their bond strength with the luting agent. However, this etchant did not etch zirconia. Thus, the AF1/AHS3 etchant is a promising candidate for the pretreatment of silica-containing CAD-CAM materials, offering a potential alternative to HF while maintaining etching effectiveness and reducing toxicity risks.

## Figures and Tables

**Table 1:** Reagents used for preparing experimental etchants.

Chemical Formula	Reagent Name, Purity	Manufacturer	Lot
NH_4_F	Ammonium fluoride, >97.0%	FujifilmWako Pure Chemical Corp, Osaka, Japan	WTJ1617
NH_4_HSO_4_	Ammonium hydrogen sulfate, >98.0%	Fujifilm Wako Pure Chemical Corp, Osaka, Japan	LEQ2979

**Table 2:** Composition of the experimental etchants prepared from ammonium fluoride (AF) and ammonium hydrogen sulfate (AHS).

Experimental Etchant Name	Weight (g)			Weight Ratio
	AF	AHS	H_2_O	AF:AHS
AF	0.45	0	8.55	1:0
AF9/AHS1	0.45	0.05	8.55	9:1
AF3/AHS1	0.45	0.15	8.55	3:1
AF1/AHS1	0.45	0.45	8.55	1:1
AF1/AHS3	0.45	1.35	8.55	1:3
AF1/AHS9	0.45	4.05	8.55	1:9
AHS	0	4.05	8.55	0:1

**Table 3:** Commercial hydrofluoric acid (HF) etchant and luting agent (adhesive primer and resin cement).

Material Type	Product Name	Manufacturer	Composition	Lot
Hydrofluoric acid (HF)	Porcelain Etchant	Bisco, Inc., Itasca, IL, America	Buffered 9.5% hydrofluoric acid	8G0249
Adhesive primer	Ceramic Primer Plus	Kuraray Noritake Dental, Tokyo, Japan	Ethanol, γ-MPTS, MDP	2200004805
Resin cement	Panavia V5	Kuraray Noritake Dental, Tokyo, Japan	Bis-GMA, TEGDMA, titanium dioxide	3P0075

Bis-GMA: bisphenol A-glycidyl methacrylate. TEGDMA: triethylene glycol dimethacrylate. γ-MPTS: 3-methacryloxypropyl trimethoxy silane. MDP: 10-methacryloyloxydecyl dihydrogen phosphate.

**Table 4:** CAD-CAM materials used in this study. The compositions are referred to as manufacturer’s information.

Material Type	Product Name	Manufacturer	Composition	Lot
Lithium disilicate glass	IPS e.max CAD	Ivoclar Vivadent, Schaan AG, Liechtenstein	57–80% SiO_2_, 11–19% Li_2_O, 0–13% K_2_O, 0–11% P_2_O_5_, 0–8% ZrO_2_, 0–8% ZnO, 0–5% Al_2_O_3_, 0–5% MgO, 0–8% coloring oxides	ZO40C6
Feldspathic porcelain	VitaBloc Mark II	Vita Zahnfabrik, Bad Säckingen, Germany	55–70% SiO_2_, 20–24% Al_2_O_3_, 6–10% Na_2_O, 4–8% K_2_O, 0–1% CaO, 0–1% TiO_2_, 0–1% pigments	98170
Polymer-infiltrated ceramic network	Vita Enamic	Vita Zahnfabrik, Bad Säckingen, Germany	58–63% SiO_2_, 20–23% Al_2_O_3_, 9–11% Na_2_O, 4–6% K_2_O, 0.5–2% B_2_O_3_, 0–1% ZrO_2_, 0–1% CaO, 14% polymer	82820
Resin composite	Cerasmart 300	GC Corporation, Tokyo, Japan	Barium glass, silica, Bis-MEPP, UDMA	1712141
Zirconia	IPS e.max ZirCAD	Ivoclar Vivadent, Schaan AG, Liechtenstein	88–95.5% ZrO_2_, 4.5–6% Y_2_O_3_, 0–5% HfO_2_, 0–1% Al_2_O_3_, 0–1% coloring oxides	ZO2GBK

**Table 5:** Failure modes for the material/cement interface following the shear bond strength tests.

Material	Treatment	No Thermocycles		20,000 Thermocycles	
		Adhesive	Cohesive	Adhesive	Cohesive
Lithium disilicate glass	No treatment	7	0	7	0
	AF1/AHS3	7	0	7	0
	HF	7	0	7	0
Feldspathic porcelain	No treatment	0	7	0	7
	AF1/AHS3	0	7	0	7
	HF	0	7	0	7
Polymer-infiltrated ceramic network	No treatment	0	7	4	3
	AF1/AHS3	0	7	0	7
	HF	0	7	0	7
Resin composite	No treatment	7	0	7	0
	AF1/AHS3	7	0	7	0
	HF	7	0	7	0
Zirconia	No treatment	7	0	7	0
	AF1/AHS3	7	0	7	0
	HF	7	0	7	0

## Data Availability

Data are contained within article.
